# Self-concept mediates the relationships between childhood traumatic experiences and adolescent depression in both clinical and community samples

**DOI:** 10.1186/s12888-024-05671-w

**Published:** 2024-03-26

**Authors:** Yufei Hu, Ying Yang, Zhengna He, Duanwei Wang, Feiyu Xu, Xingxing Zhu, Kangcheng Wang

**Affiliations:** 1 School of Psychology, Shandong Normal University, 250358 Jinan, China; 2 Shandong Mental Health Center, 250014 Jinan, China; 3 Department of Psychiatry, School of Clinical Medicine, Cheeloo College of Medicine, Shandong University, 250012 Jinan, China; 4 School of Health and Wellbeing, University of Glasgow, G12 8TB Glasgow, UK

**Keywords:** Adolescent depression, Childhood trauma, Self-concept, Chain mediation

## Abstract

**Background:**

Childhood trauma is a pivotal risk factor for adolescent depression. While the association between childhood trauma and depression is well-established, the mediating role of self-concept has not been acknowledged. Specifically, limited attention has been paid to how childhood maltreatment impacts adolescent depression through physical and social self-concept, both in clinical and community samples. This study aims to investigate how distinct and cumulative childhood trauma affects adolescent depression, as well as the potential mediating role of self-concept in their relationships.

**Methods:**

We recruited 227 depressed adolescents (dataset 1, 45 males, age = 15.34 ± 1.96) and 574 community adolescents (dataset 2, 107 males, age = 16.79 ± 0.65). Each participant was assessed on five subtypes of childhood trauma severity, cumulative trauma index, physical and social self-concept, and depression. Mediation models were tested separately in the clinical and community samples.

**Results:**

Clinically depressed adolescents experienced a higher level of trauma severity, a greater number of trauma subtypes, and had lower levels of physical and social self-concept compared to community adolescents. Analyses on childhood trauma severity and cumulative trauma index jointly indicated that physical and social self-concept played mediation roles in the relationships between childhood trauma experiences and depression. Moreover, the mediating effects of self-concept were stronger in depressed adolescents when compared to community samples.

**Conclusions:**

Our findings suggest that physical and social self-concept play mediating roles in the pathway linking childhood trauma and adolescent depression, particularly in clinically depressed individuals.

**Supplementary Information:**

The online version contains supplementary material available at 10.1186/s12888-024-05671-w.

## Introduction

Adolescent depression is a severe global mental health issue that greatly affects individuals between the ages of 10 and 19 [1]. It is one of the primary causes of disability and suicide in this age group [2], particularly after the COVID-19 pandemic [3, 4]. Adolescents with depression experience a range of symptoms, including anhedonia, guilt, low self-worth, sadness, disturbed sleep, and difficulty in concentrating. These symptoms have a significant negative impact on their academic performance and social interactions [5, 6]. Early-onset depression during this stage increases the likelihood of experiencing psychiatric and brain dysfunction later in adulthood [7]. Therefore, it is crucial to identify early-life risk factors for adolescent depression. Increasing evidence suggests that susceptibility factors from the family, peer, and childhood environments may contribute to adolescent depression. A recent behavioral network analysis has emphasized the importance of early childhood environments in comparison to other family and peer factors [8]. Understanding how adverse childhood experiences may hinder individuals’ development and increase their vulnerability to adolescent depression is necessary to identify potential interventions for those at higher risk.

Childhood trauma is a common outcome of exposure to adverse childhood experiences and significantly increases the risk of developmental issues in adolescence and adulthood [9, 10]. Childhood trauma encompasses various forms, such as emotional abuse, physical abuse, sexual abuse, emotional neglect and physical neglect [11]. Numerous studies have examined the impact of childhood trauma on depression, with consistent findings showing that more experiences of childhood trauma are associated with more severe depressive symptoms [12–14]. Three distinct models, each with different conceptual and statistical approaches, have been proposed for childhood trauma [15]: cumulative risk, individual risk (specificity), and the Dimensional Model of Adversity and Psychopathology [16]– the last one is beyond the scope of this study as it focuses on neurobiological mechanisms. The cumulative risk model emphasizes the number of trauma exposures and captures the additive effects of concurrent traumas [17], presuming a dose-response relationship where the quantity, not the specific nature, of adverse experiences shapes outcomes. For instance, it assumes an individual with physical and sexual abuse history is affected similarly to an individual with neglect and poverty history. However, it cannot distinguish whether distinct trauma subtypes have unique associations with clinical outcomes [18]. On the other hand, individual risk models prioritize the type of trauma exposure, aiming to elucidate how specific subtypes (e.g., sexual abuse vs. physical abuse) distinctly link with potential outcomes [19]. Yet, individual risk models overlook the co-occurrence of multiple traumas and may overstate the relationship between a single subtype and depression. Therefore, examining both cumulative risk and individual risk models together would provide a more comprehensive understanding of these associations, allowing the scrutiny of impacts of both aggregated and specific trauma subtype exposures. Thus, the current study not only focuses on individual trauma severity, but also considers the cumulative number of trauma types experienced by participants, and then explores their effects on adolescent depression [20–22].

Cognitive models of depression posit that environmental triggers can activate self-referential schemas associated with depression, leading to biased thinking and depressive symptoms [23]. Thus, self-concept may serve as a crucial mediator linking childhood trauma and depression [24, 25]. Self-concept is an essential component of individuals’ cognitive system and plays a role in regulating ongoing behaviors [26, 27]. It represents the image we have of ourselves [28]. Adolescence is a critical period for developing independence, self-concept and identity exploration [29]. Individuals who have experienced childhood traumatic events are more likely to develop dysfunctional attitudes, which can affect information encoding and processing during adolescence. When faced with adverse life events, individuals may develop a negative self-concept, which can directly contribute to the onset of depression [23]. Both cross-sectional and longitudinal studies have shown that negative self-concept mediates the relationship between childhood adversities and internalizing/externalizing problems in children and adolescents [24, 30, 31]. However, previous studies have mainly focused on non-clinical samples [20, 32, 33], and the mechanisms through which childhood trauma affects depression may vary in clinically depressed adolescents due to variations in emotional regulation ability, social support, and even brain development trajectories [34, 35]. It remains unclear whether self-concept plays an equivalent role in clinically depressed adolescents. Additionally, prior research has primarily examined the mediating role of general self-concept [24, 27], necessitating further research to explore how specific subtypes of self-concept function in this pathway.

During adolescence, it is crucial to form a stable and positive self-concept as it sets the stage for later developmental milestones in life. Adolescents rapidly develop their self-concept through cognitive advancements, perspective taking, and social comparison [36]. One important construct of self-concept is the physical self-concept, which emerges in childhood and undergoes significant development during adolescence. It refers to how individuals perceive their physical appearance [37]. A weak and negative physical self-concept may reduce adolescents’ physical activity, leading to a negative self-image and an increased risk of emotional dysregulation [38]. For adolescents, another crucial construct of self-concept is the social self-concept. It reflects how individuals perceive their ability and social skills in interacting with peers. Social self-concept emerges later than physical self-concept, as the development of physical abilities and appearance typically precedes individuals’ social comparison [39]. An unhealthy social self-concept also contributes to the development of internalizing problems, including depression [40]. During the development of self-concept in adolescence, early life adversities or childhood trauma may increase the risk for the development of negative self-concepts [25], which subsequently increase the risk of depression [41]. One possible explanation for this is that negative experiences may influence adolescents’ neural sensitivity to social cues [40]. Taken together, existing research suggests that both physical and social self-concept may mediate the relationship between childhood traumatic experiences and adolescent depression.

To summarize, although many studies have explored the link between childhood trauma and depression, there is limited research on how specific self-concept constructs, such as physical and social self-concept, act in their associations. Furthermore, it remains unclear whether these crucial self-concept constructs play an equivalent role in clinical adolescents diagnosed with depression. Therefore, the aim of this study is to examine the associations among childhood trauma, physical self-concept, social self-concept, and adolescent depression. Specifically, the study investigates whether physical self-concept and social self-concept mediate the relationships between childhood trauma and adolescent depression, and whether these mediating effects are present in both clinical and community adolescents.

## Methods

### Participants

Dataset 1 included 227 adolescents with a current or past diagnosis of major depressive disorder (MDD, 45 males, age = 15.34 ± 1.96, age range, 9.00–19.40). All of them were recruited from the Shandong Mental Health Center and were diagnosed with MDD according to the DSM-5 criteria. Exclusion criteria for these patients were as follows: (1) a current or past diagnosis of schizophrenia, bipolar disorder, borderline personality disorder, autism, or attention deficit hyperactivity disorder; (2) incomplete or invalid data on any of the psychological measurements; and (3) the absence of accompanying family members during the survey.

Dataset 2 included a community sample of 574 adolescents (107 males, age = 16.79 ± 0.65, age range, 14.50–20.18). These participants were recruited from local high schools in the Shandong province. The inclusion criteria of this group were as follows: (1) no current or past diagnosis of any psychiatric disorders such as MDD, schizophrenia, or bipolar disorder; (2) no history of mental disorders among any first-degree relatives; and (3) complete data on all psychological measurements.

This study only included individuals who are not lesbian, gay, bisexual, or transgender (LGBTQ). All participants (dataset 1 and dataset 2) in this study were Chinese and belonged to the Han ethnic group. This study received approval from the local ethics committee at Shandong Normal University, and written or electronic informed consent was provided by their parents.

### Psychological measurements

#### Childhood trauma

Childhood trauma was assessed using the self-report Chinese version of the Childhood Trauma Questionnaire [42]. This scale consisted of 28 items that measure 5 distinct subtypes of trauma during their childhood: emotional neglect, physical neglect, sexual abuse, emotional abuse, and physical abuse [11]. Each item was rated on a 5-point scale, ranging from “never” (score = 1) to “always” (score = 5). The total score and 5 subscale scores were calculated by the sum of corresponding items and reflected the severity of general and individual type of maltreatment. Higher scores indicated a greater extent of exposure to abuse or neglect experience. The Chinese version of this scale has been proven to be reliable and valid in Chinese adolescents [42]. The Cronbach’s alpha coefficients in dataset 1 and dataset 2 were 0.87 and 0.93, respectively.

We also calculated the cumulative risk of childhood trauma and referred it as the childhood trauma index (CTI), in line with prior studies [20, 22, 43]. The CTI reflected the cumulative number of moderate or severe trauma types, according to guidelines from the CTQ manual [44] and previous studies [45–47]. Specifically, participants who met the following criteria for each subtype were considered to have experienced the corresponding subtype of childhood trauma: emotional abuse score ≥ 13, physical abuse score ≥ 10, sexual abuse score ≥ 8, emotional neglect score ≥ 15, or physical neglect score ≥ 10. The CTI was then calculated by summing the number of these subtypes experienced by participants. Thus, the CTI could serve as an index representing the cumulative number of different types of childhood trauma experiences for each participant.

#### Adolescent depression

The severity of depression was assessed using the Child Depression Inventory [48, 49]. This inventory consisted of 27 items, each describing a certain depressive symptom. The total score ranged from 0 to 54, with higher scores indicating a greater degree of depression. The Chinese version of this inventory has been proven to be reliable and valid in Chinese adolescent population [48, 50]. In our dataset 1 and 2, the Cronbach’s alpha coefficients for the inventory were 0.87 and 0.90, respectively. We further defined four levels of depression according to YR Bang, JH Park and SH Kim [51], with scores ≥ 15 and < 20 representing mild depression, scores ≥ 20 and < 25 indicating moderate depression, scores ≥ 25 indicating severe depression, and scores lower than 15 indicating no depression.

#### Self-concept

The physical and social self-concepts were measured using the respective subscales of Tennessee Self-Concept Scale [52, 53]. Participants rated each item on a 5-point scale ranging from “completely inconsistent” (score = 1) to “completely consistent” (score = 5). Higher scores indicated a more positive level of physical and social self-concept [53]. The Cronbach’s alpha coefficients for the whole scale, subscales of physical and social self-concept in our dataset 1 and 2 were as follows: whole scale (0.84 and 0.88), physical self-concept (0.70 and 0.79), and social self-concept (0.81 and 0.85), respectively.

### Statistical analysis

All statistical analyses were performed using SPSS 26.0. Descriptive statistics and Pearson’s correlation were performed to examine the distributions of childhood traumatic experiences, self-concept, adolescent depression, and the relationships among these variables. To examine the effect of childhood trauma severity and CTI on adolescent depression, we performed linear regression analyses in the clinical and community samples separately. In these analyses, depression severity was included as the dependent variable; CTI and five subtypes of childhood trauma were regarded as the independent variables. Age and gender were included as covariates, as prior research has revealed their significant effects on both depression and trauma [12, 54].

**Fig. 1:**
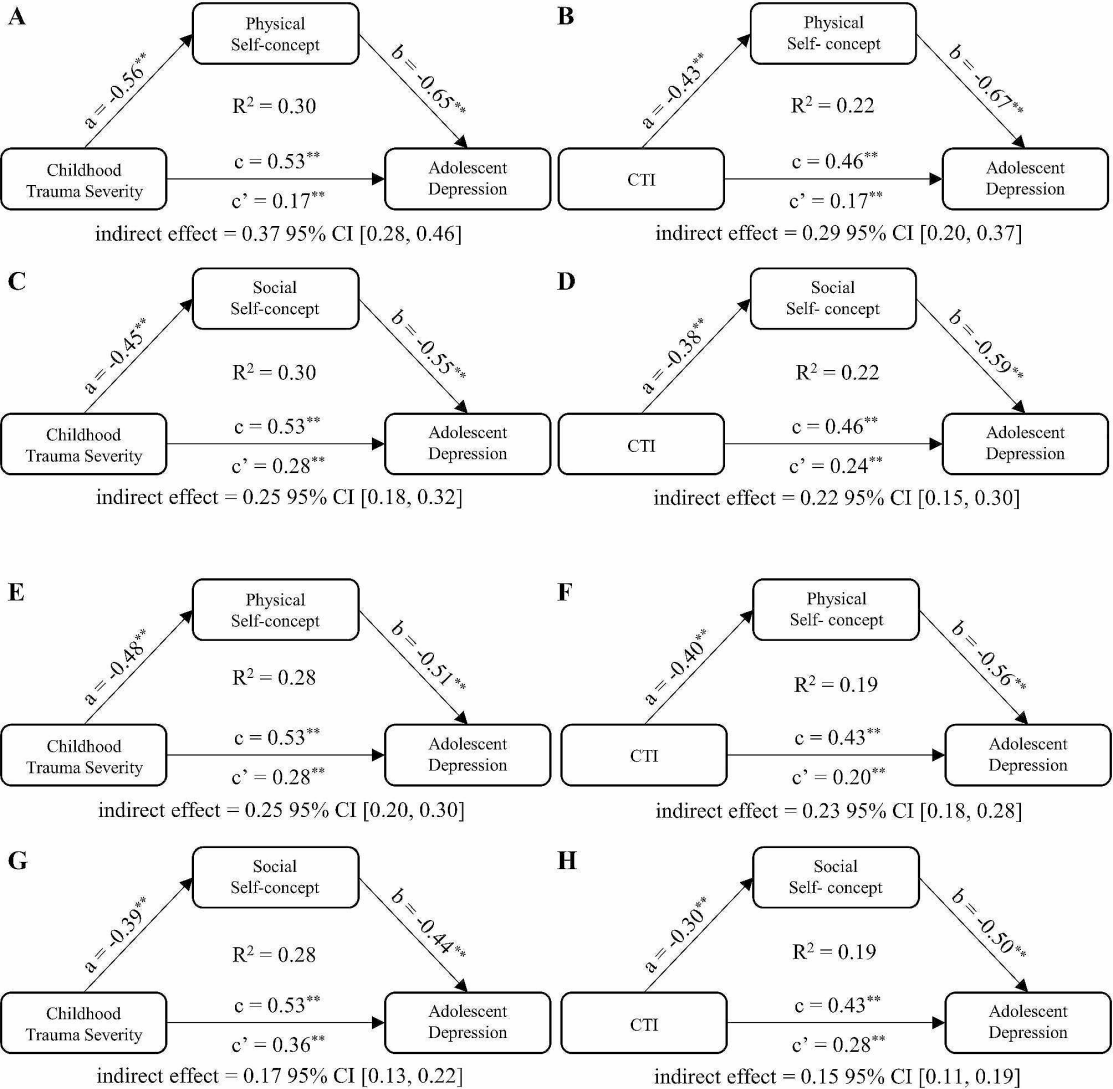
Simple mediating models test the physical and social self-concept in the relationships between childhood trauma experiences and adolescent depression. **A** to **D** represent the clinical sample (dataset 1), while **E** to **H** represent the community sample (dataset 2). In the models, both childhood trauma severity and CTI are independent variables, adolescent depression is the dependent variable, and physical self-concept and social self-concept act as mediators, respectively. All paths and mediating effects are significant (*p < 0.05; **p < 0.01)

**Fig. 2:**
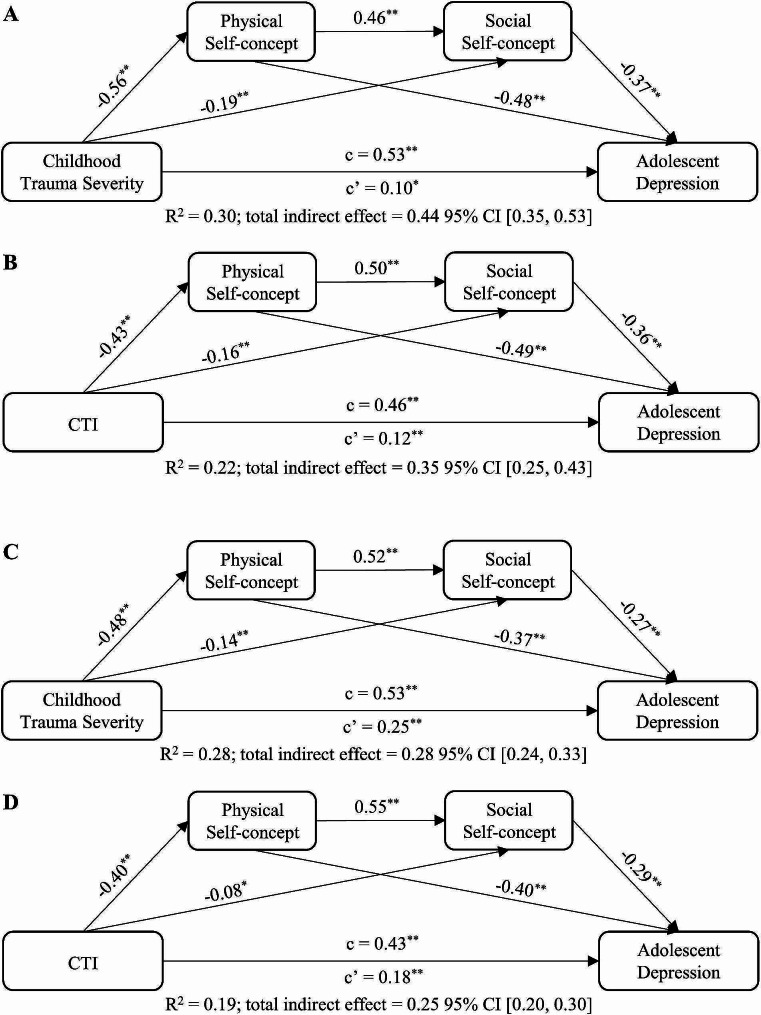
The chain mediation models of self-concept in relationships between childhood trauma experiences and adolescent depression. **A** and **B** represent the results of the clinical sample (dataset 1), while **C** and **D** represent the results of the community sample. All the paths in these models were significant (*p < 0.05; **p < 0.01)

The SPSS PROCESS macro 4.0 was utilized to conduct the mediation analyses. Firstly, simple mediation models (Fig. 1) were tested to examine whether physical or social self-concept mediated the associations between both childhood trauma severity and CTI and depression in dataset 1 and 2, respectively. Age and gender were included as covariates in all models. Given that physical self-concept has been found to emerge earlier than social self-concept and influence it [39], chain mediating models (Fig. 2) were further tested. In these models, physical self-concept was included as the first mediating variable and social self-concept was included as the second. Similarly, in the chain models, childhood trauma severity or CTI was the independent variable and adolescent depression was the dependent variable. We employed Bootstrap method to examine the significance, which estimates indirect effects and their 95% confidence intervals (CI) by computing the mean across 5000 bootstrap samples. If the CI does not include zero, it indicates the presence of statistically significant indirect effects [55].

To examine potential effects of gender ratio imbalance, we conducted two sensitivity analyses. (1) We performed same mediation analyses by randomly selecting an equal number of males and female participants (Dataset 1: 45 males and 45 females; Dataset 2: 107 males and 107 females). (2) We separately repeated the mediation analyses by only including female participants and only male participants in both dataset 1 and dataset 2.

## Results

### Sample characteristics and preliminary analyses

The means and standard deviations for all variables were presented in Table 1. The distribution of childhood trauma and adolescent depression was presented in Table 2.

**Table 1:** Descriptive statistics of depression, childhood trauma and self-concept for the clinical and community samples

Characteristics	Clinical sample			Community sample	
	Mean	SD		Mean	SD
Age	15.34	1.96		16.79	0.65
Adolescent depression	23.15	11.50		16.02	7.45
Childhood trauma severity	43.93	13.27		37.42	8.31
Emotional abuse severity	9.74	4.55		7.16	2.53
Physical abuse severity	6.26	2.67		5.50	1.43
Sexual abuse severity	5.79	2.15		5.33	1.17
Emotional neglect severity	13.06	5.11		10.47	3.99
Physical neglect severity	9.07	3.23		8.97	2.83
CTI	1.26	1.36		0.68	0.87
Physical self-concept	38.28	8.37		43.52	6.21
Social self-concept	37.55	9.25		41.22	7.10

CTI: childhood trauma index; SD, standard deviation

**Table 2:** Summary of childhood traumatic experiences and depression in the clinical and community samples

Variables	Category	n		Percentage (%)	Total
		Clinical	Community		
Childhood trauma	No experience	89	300	39.21/52.26	227/574
	Experienced	138	274	60.79/47.74	
	Emotional abuse	58	20	25.55/3.48	
	Physical abuse	27	14	11.89/2.44	
	Sexual abuse	26	17	11.45/2.96	
	Emotional neglect	88	96	38.77/16.72	
	Physical neglect	88	234	38.77/40.77	
CTI	Only emotional abuse	10	1	7.25/0.36	138/274
	Only physical abuse	2	2	1.45/0.73	
	Only sexual abuse	5	8	3.62/2.92	
	Only emotional neglect	20	19	14.49/6.93	
	Only physical neglect	21	153	15.22/55.84	
	Any two subtypes	36	74	26.09/27.01	
	Any three subtypes	23	10	16.67/3.65	
	Any four subtypes	17	5	12.32/1.82	
	Five subtypes	4	2	2.90/0.73	
Adolescent depression	No depression	63	260	27.75/45.30	227/574
	Mild depression	28	145	12.33/25.26	
	Moderate depression	31	81	13.66/14.11	
	Severe depression	105	88	46.26/15.33	

*Note* the values in front of the slash represent results in the clinical sample and the values behind slash represent results in the community sample. CTI: childhood trauma index

In the clinical sample (dataset 1), emotional neglect (*Mean* = 13.06, *SD* = 5.11) was highest among the five subtypes of childhood trauma. According to the cut-off scores of trauma subtypes in CTI, a total of 138 patients (60.79%) had suffered one or more subtypes of childhood trauma. The most prevalent childhood trauma subtypes were emotional neglect (*n* = 88, 38.77%) and physical neglect (*n* = 88, 38.77%). Furthermore, among these patients, 63 of them (27.75%) were not currently depressed, 28 (12.33%) were mildly depressed, 31 (13.66%) were moderately depressed, and 105 (46.26%) were severely depressed.

In the community sample (dataset 2), emotional neglect (*Mean* = 10.47, *SD* = 3.99) was also the most severe trauma subtype. Additionally, 274 (47.74%) participants had experienced at least one subtype of childhood trauma, with physical neglect (*n* = 234, 40.77%) and emotional neglect (*n* = 96, 16.72%) being the most prevalent. According to their scores on the CDI, 260 (45.30%) were non-depressed; 145 (25.26%) were mildly depressed; 81 (14.11%) were moderately depressed; 88 (15.33%) were severely depressed. The clinical sample scored significantly higher on CTQ (*t* = 8.32, *p* < 0.001; Table 1) and CDI score (*t* = 10.36, *p* < 0.001), and scored lower on physical self-concept (*t* = -9.70, *p* < 0.001) and social self-concept (*t* = -6.04; *p* < 0.001).

The correlation matrix for all variables was provided in Table 3. In the clinical sample (dataset 1), as expected, both childhood trauma (*r* = 0.54, *p* < 0.01) and CTI (*r* = 0.47, *p* < 0.01) were positively correlated with adolescent depression. Physical (*r* = -0.74, *p* < 0.01) and social (*r* = -0.69, *p* < 0.01) self-concept were negatively correlated with adolescent depression. In addition, both childhood trauma severity and CTI were negatively correlated with physical self-concept and social self-concept (*r* = -0.57 to -0.38, *p* < 0.01). Furthermore, linear regression analyses showed that both childhood trauma severity (β = 0.53, *R*^*2*^ = 0.30, *p* < 0.001) and CTI (β = 0.46, *R*^*2*^ = 0.23, *p* < 0.001) were positively associated with adolescent depression in the clinical sample (Table 4). In the community sample (dataset 2), all the correlation and regression analyses showed similar results (Tables 3 and 4).

**Table 3:** Correlation analysis between all variables in the clinical and community samples

	1.	2.	3.	4.	5.	6.	7.	8.	9.	10	11.	12
Age, 1	1											
Gender, 2	− 0.008/0.083^*^	1										
Childhood trauma severity, 3	− 0.023/-0.055	0.108/-0.039	1									
CTI, 4	− 0.029/0.008	0.079/-0.068	0.924^**^/0.864^**^	1								
Emotional abuse severity, 5	− 0.004/-0.076	0.183^**^/0.052	0.854^**^/0.704^**^	0.757^**^/0.529^**^	1							
Physical abuse severity, 6	− 0.029/-0.103^*^	− 0.001/-0.115^**^	0.629^**^/0.537^**^	0.614^**^/0.437^**^	0.482^**^/0.385^**^	1						
Sexual abuse severity, 7	0.028/-0.023	− 0.017/-0.091^*^	0.455^**^/0.416^**^	0.452^**^/0.454^**^	0.273^**^/0.294^**^	0.343^**^/0.331^**^	1					
Emotional neglect severity, 8	0.011/-0.011	0.086/-0.005	0.819^**^/0.838^**^	0.726^**^/0.685^**^	0.601^**^/0.423^**^	0.323^**^/0.323^**^	0.131^**^/0.151^**^	1				
Physical neglect severity, 9	− 0.100/-0.016	0.063/-0.058	0.786^**^/0.679^**^	0.774^**^/0.687^**^	0.571^**^/0.259^**^	0.339^**^/0.133^**^	0.332^**^/0.163^**^	0.585^**^/0.444^**^	1			
Adolescent depression, 10	− 0.058/-0.062	0.104/0.047	0.540^**^/0.527^**^	0.468^**^/0.424^**^	0.578^**^/0.461^**^	0.193^**^/0.255^**^	0.129/0.155^**^	0.465^**^/0.439^**^	0.425^**^/0.323^**^	1		
Physical self-concept, 11	0.004/0.030	− 0.165^*^/-0.068	− 0.574^**^/-0.479^**^	− 0.438^**^/-0.394^**^	− 0.605^**^/-0.425^**^	− 0.285^**^/-0.209^**^	− 0.077/-0.159^**^	− 0.495^**^/-0.391^**^	− 0.438^**^/-0.302^**^	− 0.744^**^/-0.647^**^	1	
Social self-concept, 12	0.009/0.018	− 0.096/-0.102^*^	− 0.458^**^/-0.384^**^	− 0.382^**^/-0.292^**^	− 0.431^**^/-0.312^**^	− 0.121/-0.071	− 0.071/-0.039	− 0.450^**^/-0.394^**^	− 0.416^**^/-0.242^**^	− 0.686^**^/-0.581^**^	0.574^**^/0.590^**^	1

*Note* The values in front of the slash were results in the clinical sample and those behind slash were in the community sample. ^**^ and ^*^ indicate statistically significance *p* < 0.01 and *p* < 0.05 respectively. CTI: childhood trauma index

**Table 4:** Regression analyses of childhood traumatic experiences on adolescent depression in the clinical and community samples

	β	SE	t	p	R^2^
Childhood Trauma Severity	0.534/0.528	0.049/0.032	9.444/14.860	< 0.001/<0.001	0.296/0.284
Emotional abuse	0.497/0.304	0.195/0.123	6.434/7.302	< 0.001/<0.001	0.375/0.303
Physical abuse	− 0.125/0.048	0.273/0.211	-1.973/1.185	0.050/0.237	
Sexual abuse	− 0.014/-0.005	0.319/0.242	− 0.231/-0.123	0.818/0.903	
Emotional neglect	0.156/0.235	0.164/0.080	2.135/5.479	0.034/<0.001	
Physical neglect	0.092/0.136	0.261/0.104	1.252/3.437	0.212/0.001	
CTI	0.461/0.430	0.501/0.324	7.780/11.374	< 0.001/<0.001	0.225/0.436

*Note* The values in front of the slash were results in the clinical sample (dataset 1) and those behind slash were in the community sample (dataset 2). CTI: childhood trauma index; SE: standard error

### Testing for the mediation effect of self-concept

The simple mediating tests indicated significantly indirect effects of childhood trauma and CTI on depression through physical and social self-concept, in both the clinical (indirect effects = 0.22 to 0.37, *p* < 0.01; Fig. 1, Table S1) and community samples (indirect effects = 0.15 to 0.25, *p* < 0.01; Fig. 1, Table S1).

Tests of chain mediation models also revealed significant mediating roles of both physical and social self-concept (Fig. 2, Table S2). In the clinical sample (dataset 1), childhood trauma severity was negatively associated with physical (β = -0.56, *p* < 0.01; Fig. 2, Table S2, Model A) and social (β = -0.19, *p* < 0.01) self-concept, and had significant direct effects on adolescent depression (*c’* = 0.10, *p* < 0.05). Physical (β = -0.48, *p* < 0.01) and social self-concept (β = -0.37, *p* < 0.01) were significantly associated with adolescent depression. Similarly, negative associations between CTI and physical (β = -0.43, *p* < 0.01; Fig. 2, Model B) and social (β = -0.16, *p* < 0.01) self-concept were observed; the direct effect of CTI on adolescent depression was significant (*c’* = 0.12, *p* < 0.01). Physical (β = -0.49, *p* < 0.01) and social self-concept (β = -0.36, *p* < 0.01) were significantly associated with adolescent depression.

In these two models of the clinical sample (Fig. 2, Table S2, model A and B), the total indirect effects of childhood trauma severity (indirect effect = 0.44, *SE* = 0.05, 95% CI = [0.35, 0.53]) and CTI (indirect effect = 0.34, *SE* = 0.05, 95% CI = [0.25, 0.43]) on adolescent depression through physical self-concept and social self-concept were both significant. More specially, childhood trauma experiences (childhood trauma severity and CTI) could influence adolescent depression through three pathways: (a) Childhood trauma experiences → Physical self-concept → Adolescent depression, (b) Childhood trauma experiences → Social self-concept → Adolescent depression, and (c) Childhood trauma experiences → Physical self-concept → Social self-concept → Adolescent depression. The total indirect effects in Model A and Model B accounted for 83.02% and 73.91% of the total effects respectively, indicating physical and social self-concept could partially mediate the relationship between childhood trauma severity/CTI and adolescent depression.

In the community sample (dataset 2), chain mediation models also observed significant mediating roles of both physical and social self-concept in the associations between childhood trauma severity (Fig. 2, Table S2, Model C) and CTI (Fig. 2, Table S2, Model D) and adolescent depression. The pathways from childhood trauma severity and CTI to adolescent depression mirrored those in the clinical sample. The total indirect effects of both physical and social self-concept were 0.28 (Fig. 2, Table S2, Model C) and 0.25 (Fig. 2, Table S2, Model D), account for 52.83% and 58.14% of the total effects respectively, indicating that the mediating effect sizes in clinical sample were higher than those in the community sample.

For the severity of 5 trauma subtypes in chain mediation models, we also observed that both physical and social self-concept significantly mediated their relationships with adolescent depression in both clinical and community samples (Figure S1 and Table S3).

### Sensitivity analyses of mediation effect

We firstly randomly selected an equal number of males and female participants in both clinical and community samples (dataset 1: 45 males, 45 females; dataset 2: 107 males, 107 females) and repeated the simple and chain mediation analyses. Similar results were found (simple mediation models, dataset 1 and 2, Figure S2; chain mediation models, dataset 1 and 2, Figure S3), revealing that both physical and social self-concept could mediate the associations between childhood trauma severity and CTI and adolescent depression. Given the very little difference in results from the whole clinical or community sample and the sex-balanced sample, our findings are not sensitive to the differences in numbers of males and females.

We also tested the mediation effects in female participants only for both datasets separately (dataset 1: clinical depressed adolescents, *n* = 182, age = 15.33 ± 2.01; dataset 2, community sample, *n* = 467, age = 16.81 ± 0.65). Similarly, significant mediating roles of both physical and social self-concept were found in both samples (simple mediation models, Figure S4; chain mediation models, Figure S5). For male participants only for both datasets (dataset 1: clinical depressed adolescents, *n* = 45, age = 15.37 ± 1.75; dataset 2, community sample, *n* = 107, age = 16.68 ± 0.64), we observed similar significant mediating roles of both physical and social self-concept (simple mediation models, Figure S6; chain mediation models, Figure S7).

## Discussion

Our study investigated the mechanisms underlying the associations between childhood trauma experiences and adolescent depression in both clinically depressed and community adolescents. We found that clinically depressed adolescents experienced more subtypes of childhood trauma and exhibited lower physical and social self-concept compared to community adolescents. Our analyses of childhood trauma severity and the cumulative trauma index convergingly revealed that physical and social self-concept exhibited chain mediation effects in the relationships between childhood trauma experiences and adolescent depression. Moreover, both physical and social self-concepts were found to play greater roles in clinically depressed adolescents than in the community sample. These findings enhance our understanding of how self-concept influences the link between childhood trauma and adolescent depression, potentially informing the development of interventions to improve adolescent mental health.

Our findings indicated that depressed adolescents, compared to community adolescents, exhibited more subtypes of childhood trauma and lower physical and social self-concept, which were consistent with previous research [32]. One possible explanation for this is the insufficient social support for adolescents suffering from traumatic experiences during their self-worth development [56, 57]. Additionally, similar to previous findings [58, 59], our study found that the more subtypes of trauma individuals experienced in childhood, the more likely they were to develop depression during adolescence. Furthermore, in line with prior studies [30, 31], our study revealed that the impact of both the childhood trauma severity and cumulative trauma index on adolescent depression were mediated by physical and social self-concept. Specifically, severe childhood trauma experiences could link to negative self-concept, which, in turn, may increase the risk for depressive disorder [60]. Our findings further extended upon the existing literature by demonstrating that both physical and social self-concept mediated the relationship between childhood trauma and depression in not only non-clinical, but also clinical adolescents.

Interestingly, our chain mediation analyses suggested that childhood trauma experiences may first be linked to physical self-concept and then to social self-concept, ultimately resulting in depression. Previous research has shown that childhood trauma may increase negative self-processing by reducing brain activity in regions associated with socioemotional processes [61]. This reduction in brain activity may contribute to lower self-image, acceptance, and self-esteem, resulting in anxiety and depressive symptoms [62, 63]. Moreover, different subtypes of childhood trauma may impact diverse aspects of an individual’s development, but all of them could contribute to negative self-concepts [64, 65]. Additionally, physical self-concept had a negative effect on social self-concept, as physical appearance often leads to social discrimination [66]. Our results suggested that childhood trauma experiences may increase the risk of adolescent depression through the chain mediation effects of physical and social self-concept, which was line with the role of self-concept and social skills in the relationship between stressful experiences and depression [67]. Overall, our study suggested that clinicians or practitioners should consider fostering positive self-concept constructs encompassing both physical and social aspects to potentially alleviate depression in adolescents exposed to childhood trauma.

Furthermore, we observed that the indirect effects of the mediating analyses in the clinical sample were higher than those in the community sample. This suggested that physical self-concept and social self-concept potentially had more substantial roles in the clinical sample [68]. It is important to note that the depressed adolescents in this study have experienced more severe and types of traumas comparted to the non-clinical participants, which may result in lower self-concept and increased levels of depression. Therefore, physical and social self-concept may exhibit stronger mediating effects between childhood trauma and adolescent depression in the clinical sample.

There were some limitations that should be acknowledged. Firstly, we utilized a cross-sectional design survey, which limited our ability to determine the direction of associations among childhood trauma experiences, self-concept, and adolescent depression. It would be necessary to conduct longitudinal cohort studies to further validate these associations. Secondly, childhood trauma experiences were measured using self-report scales, which may be susceptible to individuals’ painful memories that they are unwilling to recall. This may raise the possibility of selective memories in their retrospective reports, which could potentially bias the findings [69]. Thirdly, this study included a limited number of male participants, although our validation analyses for meditation yielded similar results. Lastly, we only measured and considered physical and social self-concepts as mediating variables in the clinical and community samples. However, other constructs of self-concept, such as academic self-concept [70] and moral self-concept [71], also played crucial roles in children’s development. Therefore, future research should explore the relationships among other constructs of self-concept, such as academic and moral self-concepts, and childhood trauma experiences and adolescent depression.

## Conclusion

Our results have enhanced our understanding of the link between childhood traumatic experiences and adolescent depression, through studying both clinical and community samples of adolescents. In particular, we observed that depressed adolescents tended to experience more severe and diverse types of childhood trauma, and they also had lower levels of physical and social self-concept. Furthermore, we found that the impact of childhood trauma on adolescent depression was mediated by physical and social self-concept in a chain manner. These findings highlighted the importance of prioritizing early psychological development and promoting a positive self-concept among adolescents. Our results suggested that peers and educators should convey increased understanding and support for depressed adolescents who have experienced trauma.

### Electronic supplementary material

Below is the link to the electronic supplementary material.

Supplementary Material 1

## Data Availability

The data analyzed in the current study are available from the corresponding author on reasonable request.
