# Supplementary material for: Self-concept mediates the relationships between childhood traumatic experiences and adolescent depression in both clinical and community samples
Source: BMC Psychiatry. 2024 Mar 26;24:224. doi: 10.1186/s12888-024-05671-w (PMC10964638; doi:10.1186/s12888-024-05671-w)
Supplement: Supplementary file 1 — Supplementary Material 1 [file 12888_2024_5671_MOESM1_ESM.docx]

**Self-concept Mediates the Relationships Between Childhood Traumatic Experiences and Adolescent Depression in both Clinical and Community Samples**

Yufei Hu^1#^, Ying Yang^2,3#^, Zhengna He^1^, Duanwei Wang^2^, Feiyu Xu^2^, Xingxing Zhu^4*^, Kangcheng Wang^1,2*^

1. School of Psychology, Shandong Normal University, Jinan, 250358, China

2. Shandong Mental Health Center, Jinan 250014, China

3. Department of Psychiatry, School of Clinical Medicine, Cheeloo College of Medicine, Shandong University, Jinan 250012, China

4. School of Health and Wellbeing, University of Glasgow, Glasgow, United Kingdom

**Figure S1.** Chain mediating tests of physical self-concept and social self-concept influence the association between childhood trauma subtypes and adolescent depression.

**Figure S2.** Simple mediating models results for equal sample size of male and female participants (A, dataset 1, clinical sample: male = 45, female = 45; B, dataset 2, community sample: male = 107, female = 107).

**Figure S3.** Chain mediation models results for equal sample size of male and female participants (A, dataset 1, clinical sample: male = 45, female = 45; B, dataset 2, community sample: male = 107, female = 107).

**Figure S4.** Simple mediating models results for female participants only (A, dataset 1, clinical sample: n = 182; B, dataset 2, community sample: n = 467).

**Figure S5.** Chain mediation models results for female participants only (A, dataset 1, clinical sample: n = 182; B, dataset 2, community sample: n = 467)

**Figure S6.** Simple mediating models results for male participants only (A, dataset 1, clinical sample: n = 45; B, dataset 2, community sample: n = 107).

**Figure S7.** Chain mediation models results for male participants only (A, dataset 1, clinical sample: n = 45; B, dataset 2, community sample: n = 107)

**Table S1.** Summary statistics for simple mediating models of self-concept's influence on the association between childhood trauma and depression.

**Table S2.** Summary statistics for chain mediating models of self-concept's influence on the association between childhood trauma severity and depression.

**Table S3.** Summary statistics for chain mediating tests of self-concept's influence on the association between childhood trauma subtypes and depression.


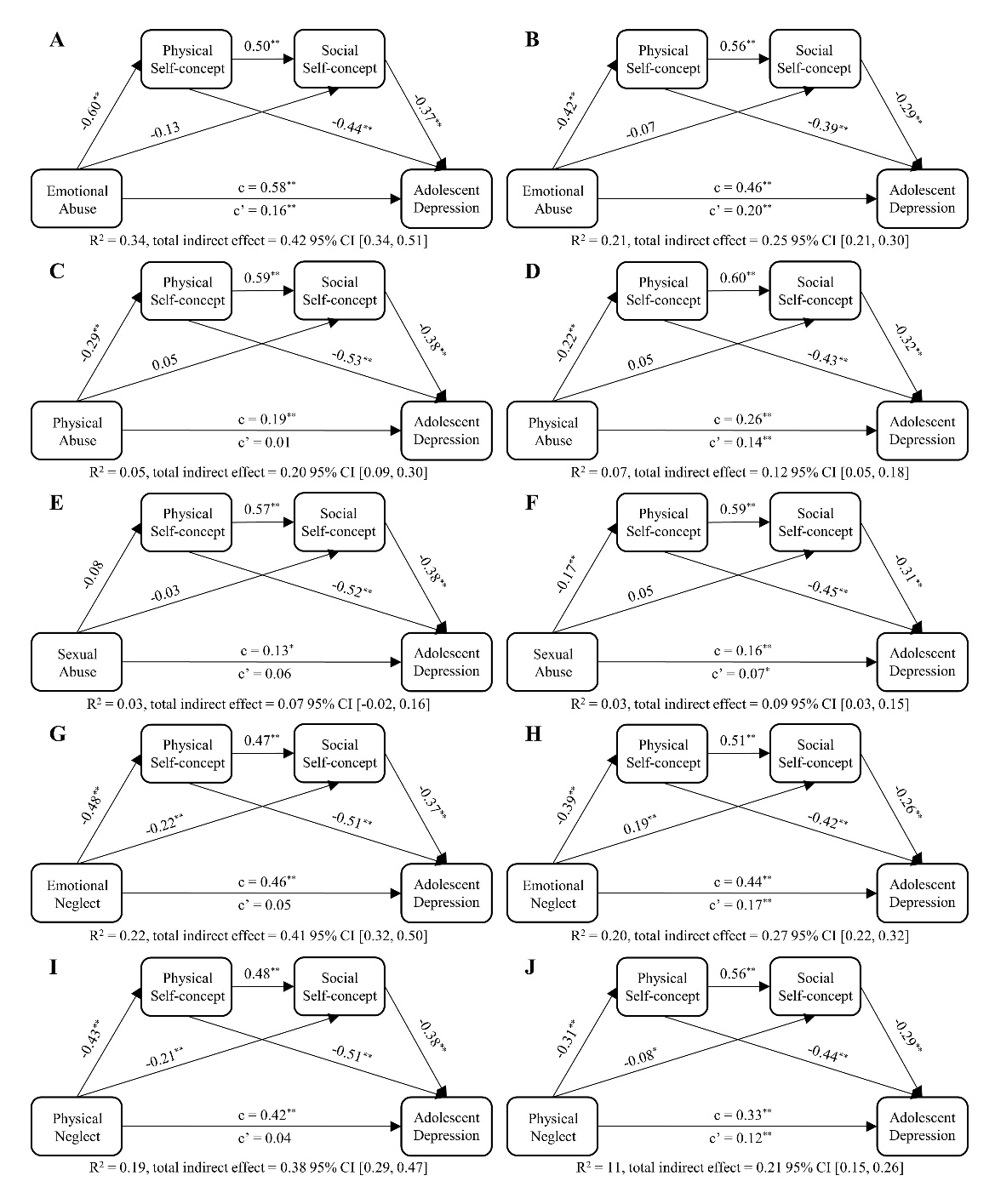


**Figure S1.** Chain mediating tests of whether physical and social self-concept influence the association between childhood trauma subtypes and adolescent depression. Left side are for the clinical sample in dataset 1 (A, C, E, G, I), right side are for the community sample in dataset 2 (B, D, F, H, J). Five subtypes of childhood trauma are independent variables respectively; adolescent depression is dependent variable; the chain from physical self-concept to social self-concept as the mediator. ^**^ and ^*^ indicate statistically significance *p* < 0.01 and *p* < 0.05 respectively.


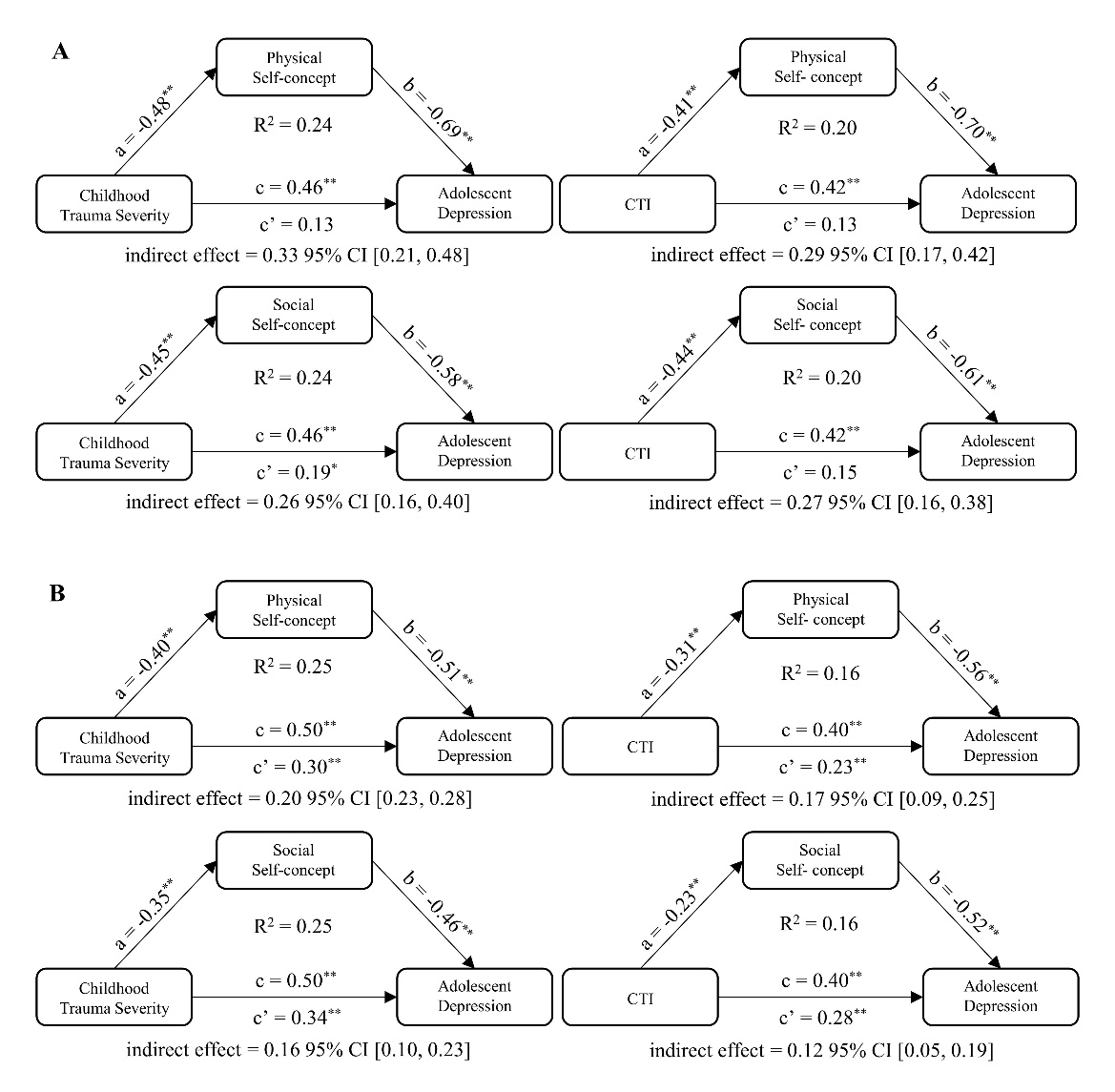


**Figure S2.** Simple mediating models in the randomly selected sample with equal number of male and female participants (A, dataset 1, clinical sample: male = 45, female = 45; B, dataset 2, community sample: male = 107, female = 107). These models examined the mediating effect of self-concept in the relationships between childhood trauma experiences and adolescent depression. In these models, all mediating effects are significant (*p < 0.05; **p < 0.01).


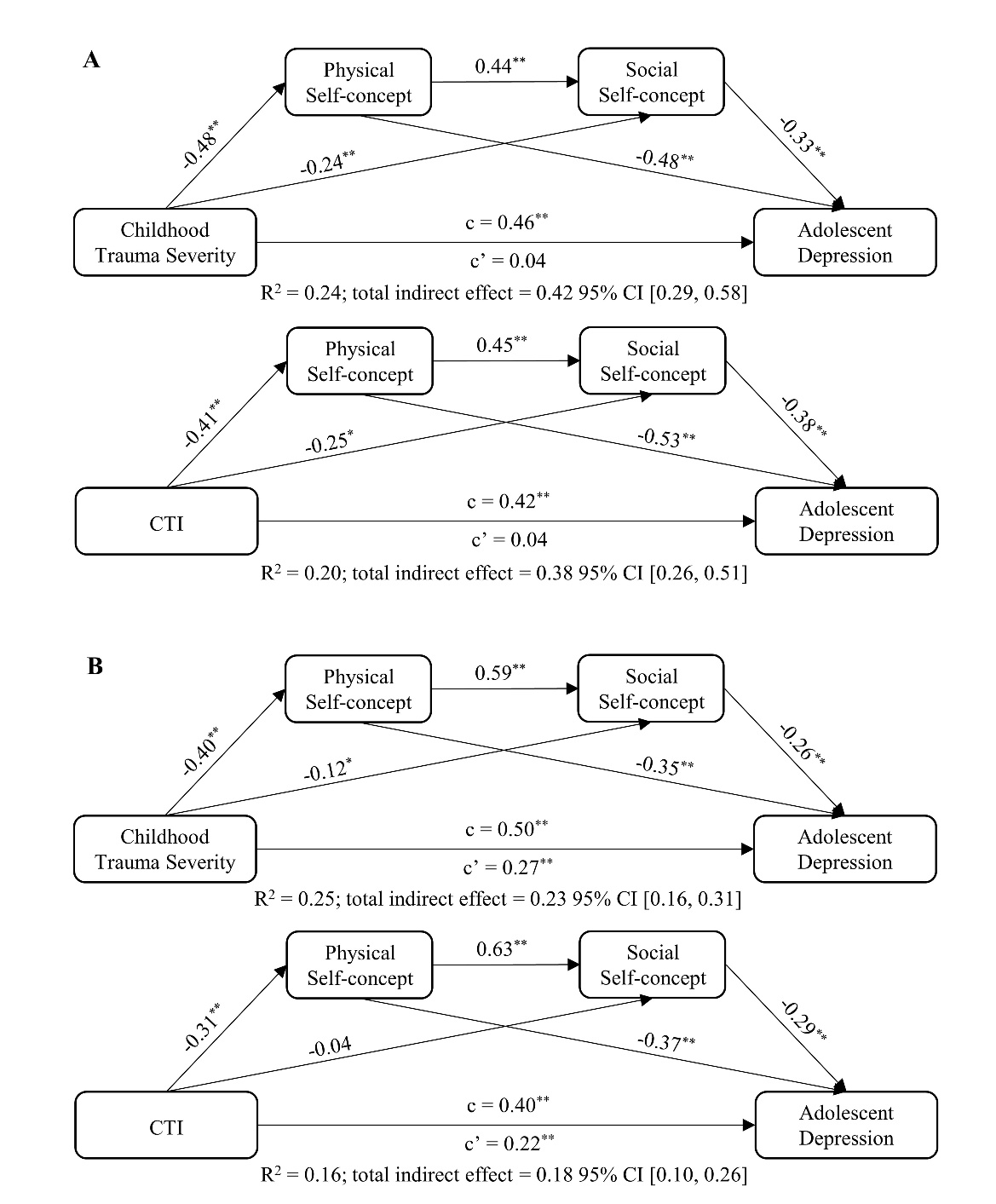


**Figure S3.** Chain mediation models in the randomly selected sample with equal number of male and female participants (A, dataset 1, clinical sample: male = 45, female = 45; B, dataset 2, community sample: male = 107, female = 107). These models examined the chain mediating effects of physical and social self-concept in the relationships between childhood trauma experiences and adolescent depression. In these models, all mediating effects are significant (*p < 0.05; **p < 0.01)


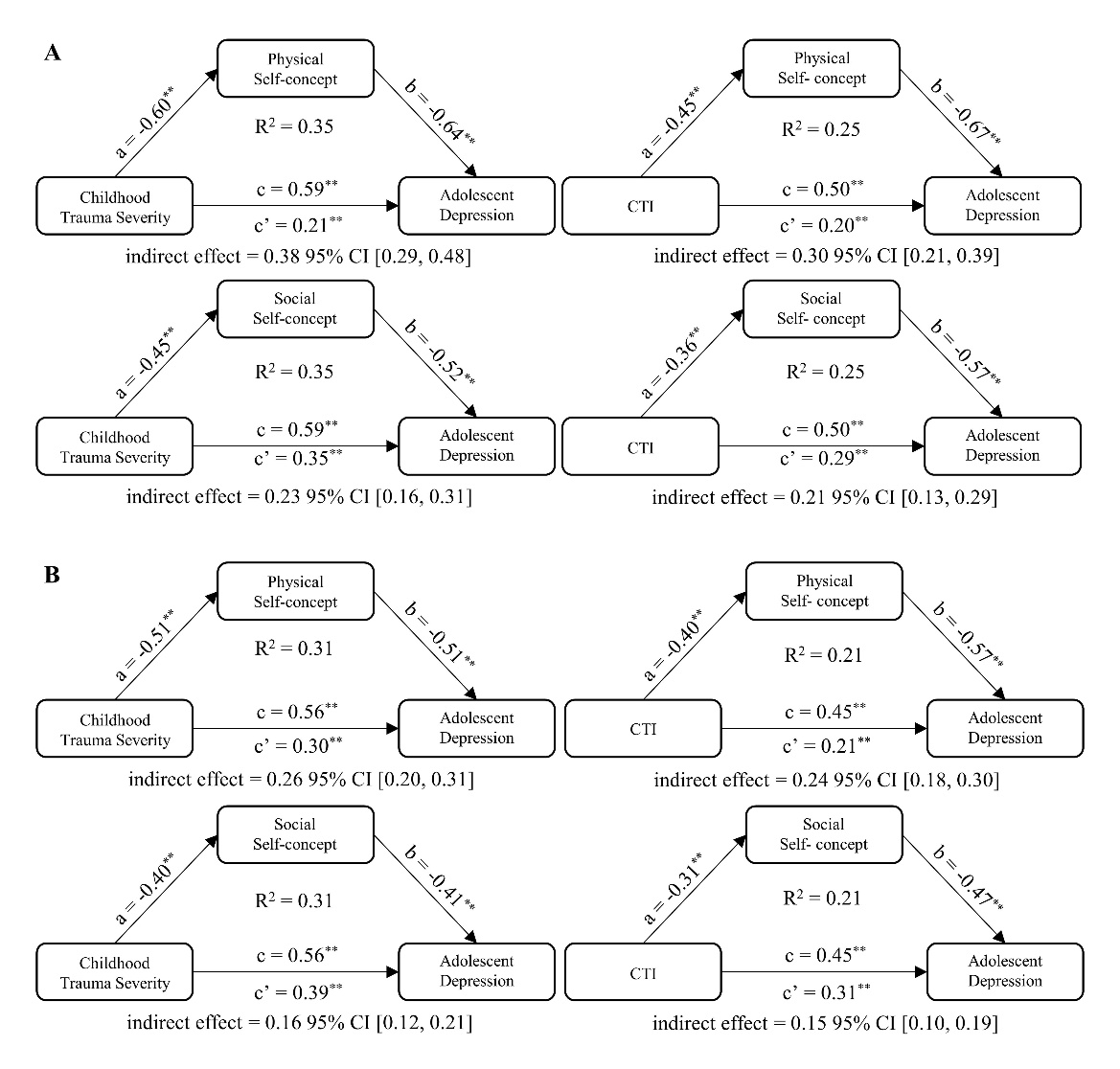


**Figure S4.** Simple mediating models in female participants only (A, dataset 1, clinical sample: n = 182; B, dataset 2, community sample: n = 467). These models examined the mediating effect of self-concept in the relationships between childhood trauma experiences and adolescent depression. In these models, all mediating effects are significant (*p < 0.05; **p < 0.01).


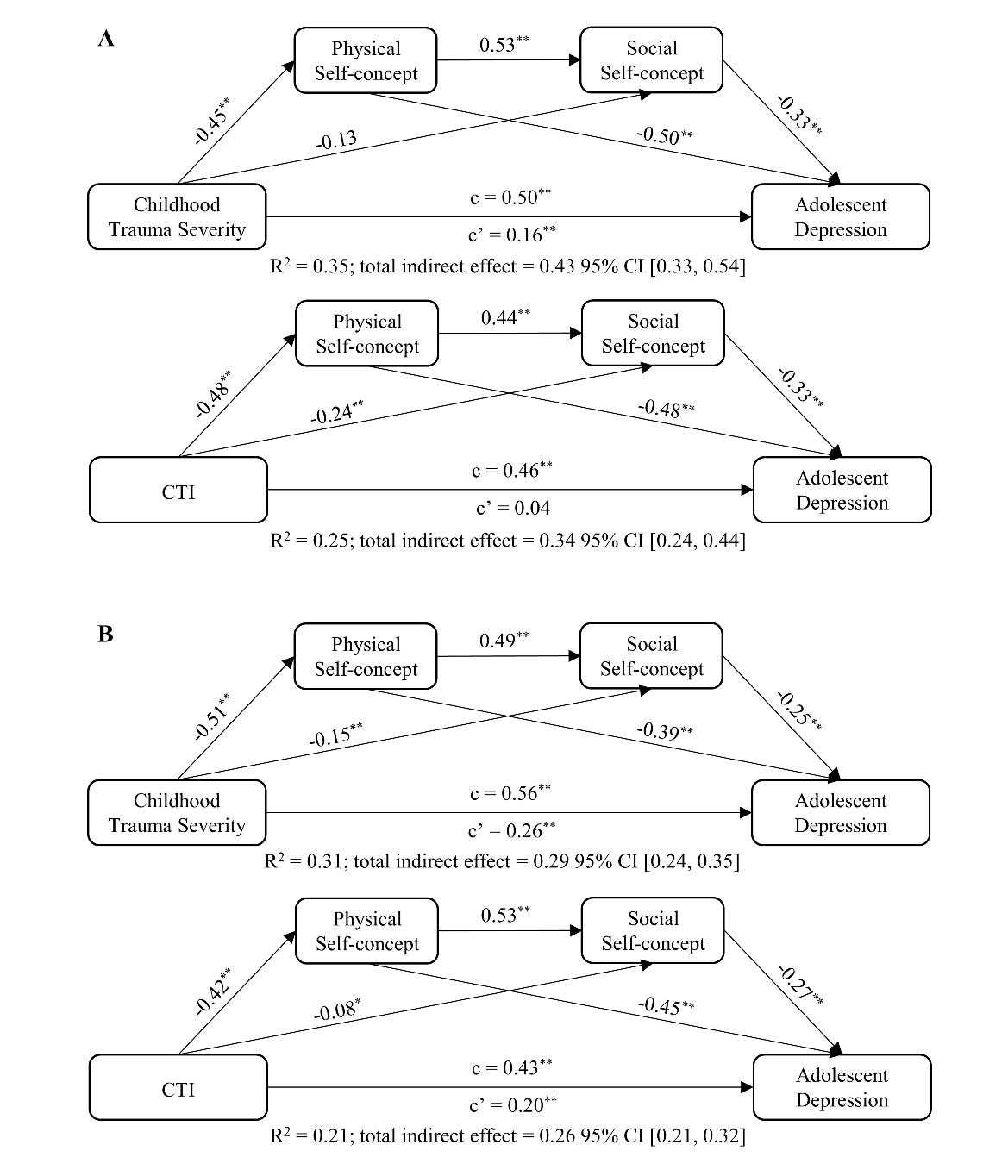


**Figure S5.** Chain mediation models in female participants only (A, dataset 1, clinical sample: n = 182; B, dataset 2, community sample: n = 467). These models examined the chain mediating effects of physical and social self-concept in the relationships between childhood trauma experiences and adolescent depression. In these models, all mediating effects are significant (*p < 0.05; **p < 0.01).


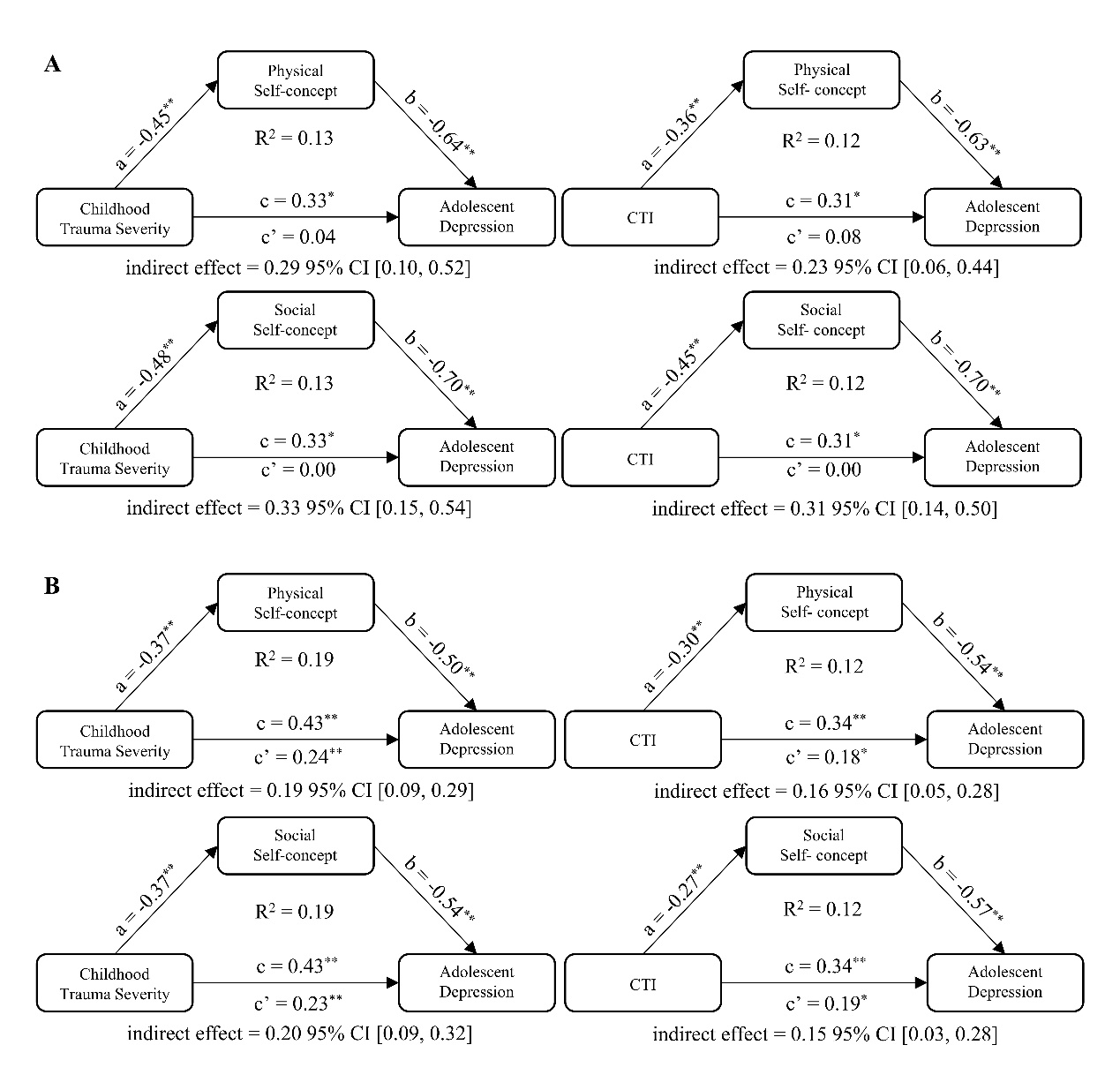
**Figure S6.** Simple mediating models results for male participants only (A, dataset 1, clinical sample: n = 45; B, dataset 2, community sample: n = 107). These models examined the mediating effect of self-concept in the relationships between childhood trauma experiences and adolescent depression. In these models, all mediating effects are significant (*p < 0.05; **p < 0.01).


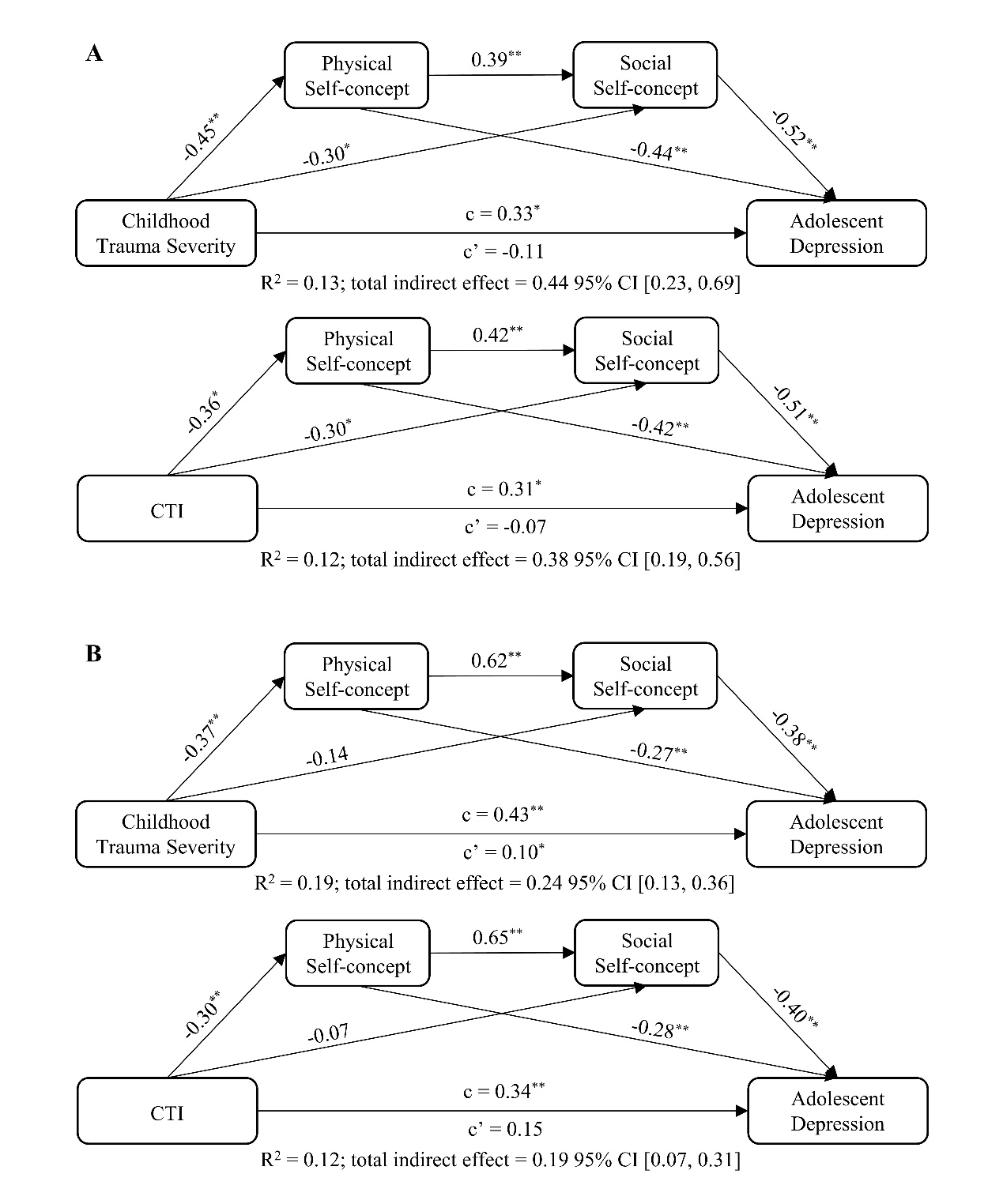
**Figure S7.** Chain mediation models results for male participants only (A, dataset 1, clinical sample: n = 45; B, dataset 2, community sample: n = 107). These models examined the chain mediating effects of physical and social self-concept in the relationships between childhood trauma experiences and adolescent depression. In these models, all mediating effects are significant (*p < 0.05; **p < 0.01).

**Table S1.** Summary statistics for simple mediating models of self-concept's influence on the association between childhood trauma and depression.

| Sample | Model in Figure 1 | | Variables and model | | R^2^ | Total (c) / direct (c') effects size | | Total indirect (ab) effects size | | SE | | 95% CI |
| --- | --- | --- | --- | --- | --- | --- | --- | --- | --- | --- | --- | --- |
| Dataset 1  Clinical sample  (N = 227) | | Model A | | Childhood Trauma Severity →  Physical Self-concept → Adolescent Depression | | 0.30 | 0.53^**^ / 0.17^**^ | | 0.37 (69.81%) | 0.04 | [0.28, 0.46] | |
|  |  | Model B | | CTI → Physical Self-concept →  Adolescent Depression | | 0.22 | 0.46^**^ / 0.17^**^ | | 0.29 (63.04%) | 0.04 | [0.20, 0.37] | |
|  |  | Model C | | Childhood Trauma Severity →  Social Self-concept → Adolescent Depression | | 0.30 | 0.53^**^ / 0.28^**^ | | 0.25 (47.17%) | 0.04 | [0.18, 0.32] | |
|  |  | Model D | | CTI → Social Self-concept →  Adolescent Depression | | 0.22 | 0.46^**^ / 0.24^**^ | | 0.22 (47.83%) | 0.04 | [0.15, 0.30] | |
| Dataset 2  Community sample (N = 574) | | Model E | | Childhood Trauma Severity →  Physical Self-concept → Adolescent Depression | | 0.28 | 0.53^**^ / 0.28^**^ | | 0.25 (47.17%) | 0.02 | [0.20, 0.30] | |
|  |  | Model F | | CTI → Physical Self-concept →  Adolescent Depression | | 0.19 | 0.43^**^ / 0.20^**^ | | 0.23 (53.49%) | 0.03 | [0.18, 0.28] | |
|  |  | Model G | | Childhood Trauma Severity →  Social Self-concept → Adolescent Depression | | 0.28 | 0.53^**^ / 0.36^**^ | | 0.17 (32.08%) | 0.02 | [0.13, 0.22] | |
|  |  | Model H | | CTI → Social Self-concept →  Adolescent Depression | | 0.19 | 0.43^**^ / 0.28^**^ | | 0.15 (34.88%) | 0.02 | [0.11, 0.19] | |

**Table S2.** Summary statistics for chain mediating models of self-concept's influence on the association between childhood trauma and depression.

| Sample | Model in Figure 2 | Variables and model | R^2^ | Total (c) / direct (c') effects size | Total indirect (ab) effects size | SE | 95% CI |
| --- | --- | --- | --- | --- | --- | --- | --- |
| Dataset 1  Clinical sample  (N = 227) | Model A | Childhood Trauma Severity → Physical Self-concept → Social Self-concept → Adolescent Depression | 0.30 | 0.53^**^ / 0.10^*^ | 0.44 (83.02%) | 0.47 | [0.35, 0.53] |
|  | Model B | CTI → Physical Self-concept → Social Self-concept → Adolescent Depression | 0.22 | 0.46^**^ / 0.12^**^ | 0.34 (73.91%) | 0.05 | [0.25, 0.43] |
| Dataset 2  Community sample (N = 574) | Model C | Childhood Trauma Severity → Physical Self-concept → Social Self-concept → Adolescent Depression | 0.28 | 0.53^**^ / 0.25^**^ | 0.28 (52.83%) | 0.02 | [0.24, 0.33] |
|  | Model D | CTI → Physical Self-concept → Social Self-concept → Adolescent Depression | 0.19 | 0.43^**^ / 0.18^**^ | 0.25 (58.14%) | 0.02 | [0.20, 0.30] |

**Table S3.** Summary statistics for chain mediating tests of self-concept's influence on the association between childhood trauma subtypes and depression.

| Sample | Model in Figure S1 | Variables and model | Direct pathways | Total (c) / direct (c') effects size | Indirect (ab) effects size | Significant indirect pathways | Each indirect pathway effect size |
| --- | --- | --- | --- | --- | --- | --- | --- |
| Clinical sample  (N = 227) | Model A | Emotional Abuse → Physical Self-concept → Social Self-concept → Adolescent Depression | Emotional Abuse → Adolescent Depression | 0.58^**^ / 0.16^**^ | 0.42 (72.41%) | Emotional Abuse → Physical Self-concept → Adolescent Depression | 0.26 (44.83%) |
|  |  |  |  |  |  | Emotional Abuse → Social Self-concept → Adolescent Depression | 0.05 (8.62%) |
|  |  |  |  |  |  | Emotional Abuse → Physical Self-concept → Social Self-concept → Adolescent Depression | 0.11 (18.97%) |
|  | Model C | Physical Abuse → Physical Self-concept → Social Self-concept → Adolescent Depression | Physical Abuse → Adolescent Depression | 0.19^**^ / 0.01 | 0.20 (105.26%) | Physical Abuse → Physical Self-concept → Adolescent Depression | 0.15 (78.95%) |
|  |  |  |  |  |  | Physical Abuse → Physical Self-concept → Social Self-concept → Adolescent Depression | 0.06 (31.58) |
|  |  |  |  |  |  | - | - |
|  | Model E | Sexual Abuse → Physical Self-concept → Social Self-concept → Adolescent Depression | Sexual Abuse → Adolescent Depression | 0.13^*^ / 0.06 | - | - | - |
|  |  |  |  |  |  | - | - |
|  |  |  |  |  |  | - | - |
|  | Model G | Emotional Neglect → Physical Self-concept → Social Self-concept → Adolescent Depression | Emotional Neglect → Adolescent Depression | 0.46^**^ / 0.05 | 0.41 (89.13%) | Emotional Neglect → Physical Self-concept → Adolescent Depression | 0.25 (54.35%) |
|  |  |  |  |  |  | Emotional Neglect → Social Self-concept → Adolescent Depression | 0.08 (17.39%) |
|  |  |  |  |  |  | Emotional Neglect → Physical Self-concept → Social Self-concept → Adolescent Depression | 0.08 (17.39%) |
|  | Model I | Physical Neglect → Physical Self-concept → Social Self-concept → Adolescent Depression | Physical Neglect → Adolescent Depression | 0.42^**^ / 0.04 | 0.38 (90.48%) | Physical Neglect → Physical Self-concept → Adolescent Depression | 0.22 (52.38%) |
|  |  |  |  |  |  | Physical Neglect → Social Self-concept → Adolescent Depression | 0.08 (19.05%) |
|  |  |  |  |  |  | Physical Neglect → Physical Self-concept → Social Self-concept → Adolescent Depression | 0.08 (19.05%) |
| Community sample (N = 574) | model B | Emotional Abuse → Physical Self-concept → Social Self-concept → Adolescent Depression | Emotional Abuse → Adolescent Depression | 0.46^**^ / 0.20^**^ | 0.25 (54.35%) | Emotional Abuse → Physical Self-concept → Adolescent Depression | 0.16 (34.78%) |
|  |  |  |  |  |  | Emotional Abuse → Physical Self-concept → Social Self-concept → Adolescent Depression | 0.07 (15.22%) |
|  |  |  |  |  |  | - | - |
|  | model D | Physical Abuse → Physical Self-concept → Social Self-concept → Adolescent Depression | Physical Abuse → Adolescent Depression | 0.26^**^ / 0.14^**^ | 0.12 (46.15%) | Physical Abuse → Physical Self-concept → Adolescent Depression | 0.09 (34.62%) |
|  |  |  |  |  |  | Physical Abuse → Physical Self-concept → Social Self-concept → Adolescent Depression | 0.04 (15.38%) |
|  |  |  |  |  |  | - | - |
|  | Model F | Sexual Abuse → Physical Self-concept → Social Self-concept → Adolescent Depression | Sexual Abuse → Adolescent Depression | 0.16^**^ / 0.07^*^ | 0.09 (56.25%) | Sexual Abuse → Physical Self-concept → Adolescent Depression | 0.08 (50.00%) |
|  |  |  |  |  |  | Sexual Abuse → Physical Self-concept → Social Self-concept → Adolescent Depression | 0.03 (18.75%) |
|  |  |  |  |  |  | - | - |
|  | Model H | Emotional Neglect → Physical Self-concept → Social Self-concept → Adolescent Depression | Emotional Neglect → Adolescent Depression | 0.44^**^ / 0.17^**^ | 0.27 (61.36%) | Emotional Neglect → Physical Self-concept → Adolescent Depression | 0.17 (38.64%) |
|  |  |  |  |  |  | Emotional Neglect → Social Self-concept → Adolescent Depression | 0.05 (11.36%) |
|  |  |  |  |  |  | Emotional Neglect → Physical Self-concept → Social Self-concept → Adolescent Depression | 0.05 (11.36%) |
|  | Model J | Physical Neglect → Physical Self-concept → Social Self-concept → Adolescent Depression | Physical Neglect → Adolescent Depression | 0.33^**^ / 0.12^**^ | 0.21 (63.64%) | Physical Neglect → Physical Self-concept → Adolescent Depression | 0.13 (39.39%) |
|  |  |  |  |  |  | Physical Neglect → Social Self-concept → Adolescent Depression | 0.02 (6.06%) |
|  |  |  |  |  |  | Physical Neglect → Physical Self-concept → Social Self-concept → Adolescent Depression | 0.05 (15.15%) |
